# Analyses of distribution and dosimetry of brain metastases in small cell lung cancer with relation to the neural stem cell regions: feasibility of sparing the hippocampus in prophylactic cranial irradiation

**DOI:** 10.1186/s13014-017-0855-3

**Published:** 2017-07-15

**Authors:** Lei Zhao, Yan Shen, Jin-Dong Guo, Heng-Le Gu, Wen Yu, Jia-Ming Wang, Chang-Xing LV, Jun Liu, Xu-Wei Cai, Xiao-Long Fu

**Affiliations:** 1 Department of Radiation Oncology, Shanghai Chest Hospital, Shanghai Jiao Tong University, No.241 West Huaihai Road, Shanghai, 200030 China; 2 Department of Radiology, Shanghai Chest Hospital, Shanghai Jiao Tong University, Shanghai, China

**Keywords:** Hippocampal avoidance, Prophylactic cranial irradiation, Brain metastases, Small cell lung cancer

## Abstract

**Background:**

This work aims to assess the feasibility of selectively sparing the hippocampus during prophylactic cranial irradiation (PCI) for small cell lung cancer (SCLC).

**Methods:**

SCLC patients with brain metastases (BMs) diagnosed with MRI were enrolled. Lesions localized to the neural stem cell (NSC) compartments [subventricular zone (SVZ) or hippocampus] were analyzed. Patients were categorized by the total number of intracranial metastases, the therapy processes and the symptoms. Hippocampi and enhanced lesions within 15 mm from the hippocampus were contoured. IMRT treatment plans were generated for hippocampal avoidance (HA)-PCI (25Gy in 10 fractions).

**Results:**

From Jan 2011 to Oct 2014, 1511 metastases were identified in 238 patients. The overall ratio of metastatic lesions located in NSC regions was 2.0% in the 1511 total metastases and 9.7% in the 238 overall patients. Among the NSC region metastases, 15 (1.0%) lesions involved the HA region of 14 (5.9%) patients and another 15 (1.0%) involved the SVZ of 15 (6.3%) patients. The involvement of HA region or SVZ was significantly different between patients with oligometastatic and non-oligometastatic BMs (*P* < 0.05). Based on the dosimetric analysis, 26 (10.9%) patients with 41 (2.7%) metastases within 15 mm from the hippocampus had inadequate dosage in case that HA-PCI was applied.

**Conclusions:**

Our retrospective review of 1511 metastases in 238 patients (among whom 89.5% were male) suggests that the metastatic involvement of the NSC regions (especially hippocampus) is unusual and limited primarily to patients with non-oligometastatic disease in SCLC. Also, dosimetric analysis shows that about 10% of patients may have adequate dosage due to HA-PCI treatment. But we believe that this is still an acceptable clinical treatment strategy for SCLC.

## Background

Prophylactic cranial irradiation (PCI) is a standard treatment for patients with limited-stage small cell lung cancer (SCLC) who have a good response to initial therapy [1]. Similarly, in patients with extensive-stage SCLC that has responded to systemic therapy, PCI decreases brain metastases (BMs) [2]. Recently, the cognitive deficits after cranial radiation attracted researchers’ attention. Brown [3] et al. reported that among patients with 1 to 3 brain metastases, the use of stereotactic radiosurgery (SRS) alone, compared with SRS combined with whole brain radiation therapy (WBRT), resulted in less cognitive deterioration at 3 months. Similar decline in Hopkins Verbal Learning Test-Revised (HVLT-R) and quality of life (QOL) has also been observed after PCI for lung cancer [4]. Although neurocognitive disorders after WBRT/PCI have a multifactorial etiology, it is currently believed that they are mostly caused by a loss of neural stem cells (NSCs) [5]. Transplantation experiments demonstrate that NSCs are restricted in the postnatal brain to regions in which they occur naturally, namely the subventricular zone (SVZ) and the subgranular zone (SGZ) of the hippocampus [6, 7]. Several exogenous and endogenous conditions negatively regulate neurogenesis in these regions, including chemotherapy, radiation therapy, glucocorticoid stress hormones and certain inflammatory states. In conclusion, avoidance of hippocampus in WBRT/PCI appears to be of essence.

Up to now, only one study with a small sample size has been carried out to estimate the risk of metastases in the hippocampal avoidance (HA) region for patients with SCLC [8]. Although existing radiotherapy technique has already certified the feasibility of sparing the hippocampus, whether HA increases the risk of inadequate dosage for BMs is unknown. The purpose of this retrospective research is to assess the feasibility of selectively sparing the hippocampus during PCI for SCLC patients and to analyze the impact of the distribution and dosimetry of BMs regarding to the NSC regions (especially hippocampus).

## Methods

### Inclusion criteria

Consecutive SCLC patients with BMs who were treated in the Radiation Oncology Clinic of Shanghai Chest Hospital from Jan 2011 to Oct 2014 were reviewed respectively. All cases were pathologically confirmed. T1-weighted, postcontrast, axial enhanced MR images dated right at the time of diagnosing BMs were required for each patient. 71.8% of the patients enrolled received WBRT sometime after diagnosis of BMs, while others did not as they refused the treatment or because of other clinical factors.

### Exclusion criteria

Patients who had received pre-treatment (radiotherapy or surgery) for brain from other diseases were excluded. Those who had been treated with PCI or pathologically diagnosed as combined SCLC were not included as well. There was no cut-off for the total number of metastases found in a single patient.

### Study design

**Fig. 1:**
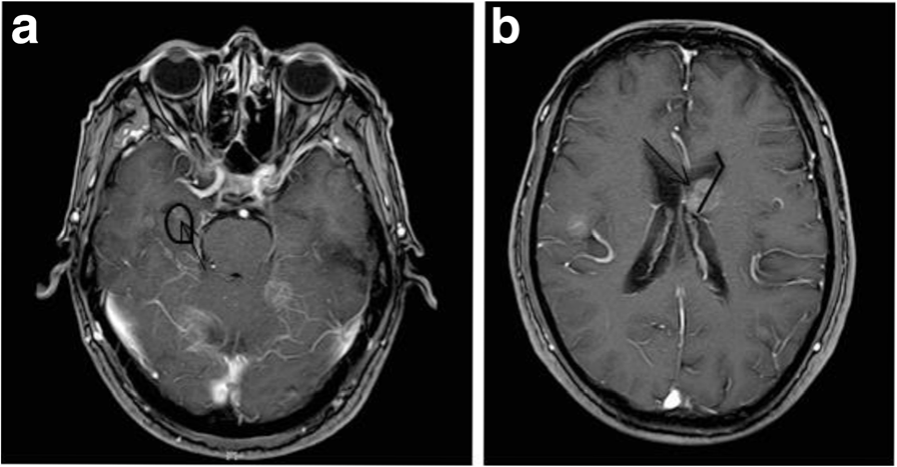
**a** Hippocampal metastasis. The *black* contour represents the hippocampal contour; the *arrow* points to the metastasis. **b** SVZ metastasis. The *black* contour represents the SVZ contour; the *arrow* points to the metastasis

T1-weighted, postcontrast, axial enhanced MR images dated right at the time of diagnosing BMs were retrieved by both a radiologist and a radiation oncologist for each patient. Axial images were used to outline the hippocampi, SVZ as well as the metastases. The anatomic boundaries of the hippocampus were identified according to the RTOG0933 hippocampal atlas [9]. Metastases in the HA region were defined as lesions within the bilateral hippocampi plus <5 mm of the surrounding area. The SVZs were defined as the 5 mm of tissue immediately adjacent to the lateral aspect of the lateral ventricle (Fig.1a and b).

The gross tumor volume (GTV) was volumetrically contoured on fused diagnostic enhanced T1 weighted MRI-planning CT images. GTV for each target metastasis was identified as the enhanced lesion within 5-15 mm from the hippocampus (peri-hippocampus) on the T1-weighted MRI. Planning target volume (PTV) was outlined using a computer-automated 3 mm 3D margin expansion of the GTV. The treatment planning system was Pinnacle^3^ treatment-planning workstation (Philips Healthcare, Andover, MA, USA; ADCA, Milpitas, CA, USA).

### Method of HA

**Fig. 2:**
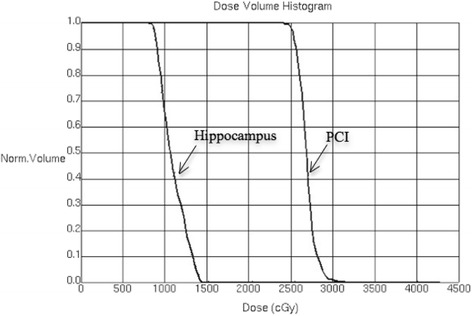
The Dose Volume Histogram of HA-PCI

To detect whether the risk of BMs in peri-hippocampus area is increased by low dose exposure caused by HA, the implementation of IMRT plans are imitated for the patients. For hippocampal contouring and HA-PCI planning, bilateral hippocampi contours were manually generated on the fused MRI-CT image set and expanded by 5 mm to generate the HA regions. The PCI clinical target volume (PCI-CTV) was defined as the whole-brain parenchyma, and the PCI-PTV was defined as the PCI-CTV excluding the HA region. IMRT was delivered to a dose of 25Gy in 10 fractions over 2 weeks to cover the PCI-PTV while avoiding the hippocampus. In HA-PCI planning protocol, a dose up to 100% of the hippocampus could not exceed 9Gy, and the maximal hippocampal dose could not exceed 16Gy. In addition, the acceptable variation for the PCI-PTV was D_95%_ ≥ 25Gy, while D_1%_ < 23.75Gy that identified as the “cold” spots was unacceptable (Fig.2).

### Statistical methods

Total metastases from all enrolled patients were assessed. All reported lesions were localized to the NSC compartments or the rest of the brain. Individual patients were categorized by the total number of intracranial metastases (oligometastatic:1–3 metastases; non-oligometastatic:4 or more metastases), the therapy processes (BM identified at diagnosis; BM identified during or after treatments) and the symptoms (symptomatic BM; asymptomatic BM). Then, the corresponding incidence of metastases in NSC regions was analyzed. Chi-square test was adopted to compare the incidence between different groups. A two-side *P* value <0.05 was used for statistical significance.

Patients who have metastases within 15 mm from the hippocampus were evaluated separately. Of these cases, the number of patients and target metastases that can not reach the acceptable variation for the PCI-PTV were recorded, including those who already have HA region involved. Then, the ratio of patients and metastases with inadequate dosage were recorded.

## Results

From Jan 2011 to Oct 2014, 238 SCLC patients with BMs were enrolled for the research. Demographic and disease-specific data of all patients are presented in Table 1. The majority of the patients were male (89.5%), smoker or former smokers (84.5%). And approximately three quarters of patients were in Recursive Partitioning Analysis (RPA) class II (74.8%).

**Table 1:** Patient characteristics (*n* = 238)

	Number (%)
Age	
> 60y	126 (52.9%)
≤ 60y	112 (47.1%)
Gender	
Male	213 (89.5%)
Female	25 (10.5%)
Smoking status	
Smoker or former smoker	201 (84.5%)
Nonsmoker	37 (15.5%)
RPA	
I	46 (19.3%)
II	178 (74.8%)
III	14 (5.9%)
Total number of intracranial metastases	
Oligometastatic	150 (63.0%)
Non-oligometastatic	88 (37.0%)
Therapy processes	
BMs identified at diagnosis	97 (40.8%)
BMs identified during or after treatments	141 (59.2%)
Symptoms	
Symptomatic BMs	65 (27.3%)
Asymptomatic BMs	173 (72.7%)

*RPA* recursive partitioning analysis, *BMs* brain metastases

The overall ratio of metastatic lesions located in NSC regions was 2.0% in the 1511 total metastases (30/1511). Among the NSC region metastases, 15 (1.0%) lesions involved the HA region of 14 (5.9%) patients and another 15 (1.0%) involved the SVZ of 15 (6.3%) patients, as Table 2 shows. 6 (2.5%) patients have the HA region and the SVZ affected simultaneously, while only 1 (0.4%) patient has bilateral hippocampi involved. Among the 14 patients with metastases in the HA region, a total of 480 lesions and 34.3 metastases per patient were observed. Meanwhile, among the 15 patients with SVZ metastases, there were a total of 480 metastatic lesions and 32 metastases per patient. So, even among the patients with a heavy burden of metastases, HA region and SVZ involvements both accounted for only 3.1% (15/480) of their total lesions.

**Table 2:** Pattern of involvements in patients with NSC region metastases and incidence of NSC lesions in different groups

	SVZ	P	Hippocampus^a^	P
No. of involved patients (%)	15^a^ (6.3%)	_	14^a^ (5.9%)	_
No. of specific metastases in involved patients (%)	15 (1.0%)	_	15^b^ (1.0%)	_
Total Number of intracranial metastases				
Oligometastatis pts	0/150 (0.0%)	0.000	1/150 (0.7%)	0.000
Non-oligometastatic pts	15/88 (17.0%)		13/88 (14.8%)	
Therapy processes				
BMs identified at Diagnosis	3/97 (3.1%)	0.109	6/97 (6.2%)	1.000
BMs identified during or after treatments	12/141 (8.5%)		8/141 (5.7%)	
Symptoms				
Symptomatic BMs	5/65 (7.7%)	0.561	1/65 (1.5%)	0.120
Asymptomatic BMs	10/173 (5.8%)		13/173 (7.5%)	

*SVZ* subventricular zone, *pts* patients, *BMs* brain metastases

^a^Including 6 patients have hippocampus and SVZ simultaneously implicated

^b^1 patient has bilateral hippocampi involved

The incidence of NSC region involved is listed in Table 2, categorized based on different clinical conditions, i.e. the total number of intracranial metastases, the therapy processes and the symptoms. Throughout the patients, the difference of the incidence of HA region or SVZ involved was significant between oligmetastatic and non-oligmetastatic patients (*P* < 0.05), but trivial for patients with different therapy processes and symptoms.

Of all patients enrolled, 36 patients had 48 additional lesions located within 15 mm from the hippocampus, except the 15 HA region involvements. Among theses 48 lesions, 26 target metastases in 18 patients can not reach the acceptable variation, i.e. PCI-PTV of D_95%_ ≥ 25Gy. As described in Table 3, totally 26 (10.9%) patients with 41 (2.7%) metastases within 15 mm from the hippocampus had inadequate dosage in our group when HA-PCI was delivered.

**Table 3:** The evaluation of underdosage in brain treated with PCI according to the different criteria of HA

Different criteria of HA		Underdosage	
		No. of patients (%)	No. of lesions (%)
Spatial distribution	≤5 mm from hippocampus	14 (5.9%)	15 (1.0%)
	≤15 mm from hippocampus	40 (16.8%)	63 (4.2%)
Dose distribution	Hippocampus: D_100_ ≤ 9Gy D_max_ ≤ 16Gy	26 (10.9%)	41 (2.7%)

*HA* hippocampal avoidance

## Discussion

Whether HA-PCI in SCLC is worthwhile or not remains unclear so far. Most studies focus on its security and feasibility. And research targeting at the reduction of normal cerebral tissue injury is still in progress. To date, several studies have investigated the incidence of metastases in HA region (Table 4) to identify the security of HA-WBRT [8–13]. However, it is worth noting that since most researchers didn’t impose restrictions on primary site of BMs, SCLC patients only accounted for a small fraction.

**Table 4:** Distribution of brain metastases in HA region for SCLC patients

Study	Kundapur et al. [8]	Gondi et al. [9]	Ghia et al. [10]	Marsh et al. [11]	Wan et al. [12]	Harth et al. [13]
No of metastatic lesions	NA	1131	272	697	2270	856
No of pts	NA	371	100	107	488	100
No of metastases in HA region (%)	NA	34 (3.0%)	9 (3.3%)	16 (2.3%)	7 (0.3%)	11 (1.3%)
No of pts. with metastases in HA region (%)	NA	22 (8.6%)	8 (8.0%)	16 (15.0%)	7 (1.4%)	11 (11.0%)
No of metastatic lesions for SCLC pts	359	NA	24	145	225	201
No of SCLC pts	59	38	10	11	44	11
No of metastases in HA region for SCLC pts. (%)	3 (0.8%)	NA	3 (12.5%)	3 (2.1%)	1 (0.4%)	5 (2.5%)
No of SCLC pts. with metastases in HA region (%)	3 (5.1%)	4 (10.5%)	3 (30.0%)	3 (27.3%)	1 (2.3%)	5 (45.5%)

*pts* patients, *HA* hippocampal avoidance, *SCLC* small cell lung cancer, *NA* not available

Even though PCI offers clinically proven safety and satisfactory therapeutic efficacy to SCLC patients, it induces adverse effects like neurocognitive deficits [14]. Consequently, HA-PCI needs to be treated with caution. In previous work, some researchers noted that the metastatic incidence of the HA region in patients with SCLC seemed to be higher than that of the others [11, 13]. Nevertheless, Gondi and colleagues [9] reported only 4 HA region metastases in 38 patients with SCLC (10.5%) and Wan et al. [12] even reported smaller rate with only 1 in 44 SCLC patients (3.0%). Such data may be skewed because of the limited sample size. Recently, Kundapur et al. [8] first assessed the risk of metastases in the HA region for SCLC patients. Of 59 patients with BMs, 3 (5.0%) had metastases in the HA region, and collectively there were 359 BMs with 3(0.8%) deposits. However, relevant data for such group were still limited. The work here founds out that the rate of involvement of the HA region to be 5.9% overall, very close to the recent research. And the incidence was not so high as described in others’ work. Up to now, this is the largest sample size study for SCLC patients, in which both patients diagnosed as combined SCLC and patients received PCI previously were excluded.

Only one study from Fudan University Shanghai Cancer Center has reviewed the metastatic involvement of the SVZ until now. Wan et al. [12] indicated that the rate of involvement of the SVZ was 0.8% (18/2270) for all intracranial metastases and 3.7% (18/488) for all patients. In our retrospective review, this rate was 1.0% (15/1511) for all intracranial metastases and 6.3% (15/238) for all patients. These results showed the incidence of BMs within the SVZ was also very low. Moreover, our work concludes that the involvement incidence of the NSC regions was lower with the oligometastatic patients than with the non-oligometastatic patients (*P* < 0.05). Similar results were also reported by Marsh [11] and Wan [12] et al. Given that the high rate of NSC regions metastases occurred to SCLC patients with non-oligometastatic disease, more attention must be paid to this subgroup. Whether HA-PCI should be performed in different strategies with certain patients need to be considered with caution.

It poses important challenges to avoid the hippocampus during cranial irradiation while allowing for uniform dose delivery to the remainder of the brain, because of the central location and the unique anatomic shape of the hippocampus. Recently, many planning studies have shown the dosimetric feasibility of HA-WBRT using different radiotherapy systems [15]. Similarly, as for BMs treatment, HA-WBRT technique can be used for PCI where the neurocognitive function (NCF) preserving approaches are even more justified. Investigators at Rush University Medical Center [16] tested the feasibility of dosimetrically sparing the hippocampus and the SVZ during helical TomoTherapy with WBRT and PCI. They finally revealed the fact that PCI differs from WBRT only in terms of fractionation and that the standard radiotherapy technique is similar. Thus, it is now technically and dosimetrically feasible to implement HA approaches into clinical practice.

At present, HA region is defined as the bilateral hippocampi plus <5 mm of the surrounding area generated on the fused MRI-CT image set in most studies including RTOG0933. A lesser dose would be applied to this defined region (HA) as compared with the remaining part of the brain. Additionally, some investigators [10] considered the area with the distance of 5 to 15 mm from the hippocampus to be the high-risk region. Metastases located in this high-risk region may have inadequate dosage in HA-PCI. To validate this problem, HA-PCI planning was implemented in patients with metastases within 15 mm from the hippocampus in our study. Ultimately, 36 patients had 48 lesions located in this high-risk area, and 26 target metastases in 18 patients can not reach the acceptable variation for the PCI-PTV. Together with the HA region involvements, 26 (10.9%) patients with 41 (2.7%) metastases had adequate dosage in HA-PCI. Yet, though it only affected 2.7% of the lesions, the proportion was over 10% for the patients. Therefore, conclusion may be drawn that about 10% of patients may have adequate dosage due to HA-PCI treatment. But we believe that this is still an acceptable clinical treatment strategy for SCLC. As relevant data have not been evaluated before, the problem needs further attention.

Another puzzling issue is that the majority of the patients enrolled were men (89.5%). SCLC had historically been seen more frequently in men than in women. However, in the latest study, Wang et al. [17] found the patient number in both sexes became similar over the last two decades due to decreasing male cases and a stable number of female cases in the United States. Similarly, Schabath et al. [18] assessed demographic characteristics of 1032 SCLC patients treated at the Moffitt Cancer Center from 1986 to 2008. The result showed 50.2% of the cases were male, which was quite different from ours. The main reason for our result is tobacco smoking [19], which is strongly associated with the development of SCLC, with only 2 to 3% of patients being never-smokers [20]. In China, the main smokers are men, who constitute the major portion of the SCLC. The percentage of women smokers is extremely low, and none of the female patients in our study has a history of smoking. So, the differences of smoking status between different races and nationalities may explain the problem.

Our data are limited by the fact that this is a retrospective, single-institution study. The statuses of BMs were not original and were affected by many factors, including the following frequency, the appearance of symptoms, etc. As shown in this research, which has the largest sample among peers’ work, the risk of BMs in NSC regions is extremely low and about 10% of patients may have adequate dosage due to HA-PCI treatment. Therefore, it is reasonable to selectively reduce dose to the hippocampus, while simultaneously to treat the remainder of the whole brain with full dose as far as possible during PCI for SCLC patients. Some problems, such as the recurrence rate of high-risk region and the preservation of cognitive function after HA-PCI, need to be further explored in the future.

## Conclusions

Our retrospective review of 1511 metastases in 238 patients (among whom 89.5% were male) suggests that the rate of involvement of the NSC regions (especially hippocampus) by intracranial metastatic disease in SCLC is very low and is limited primarily to non-oligometastatic disease with a heavy tumor burden. Also, dosimetric analysis shows that about 10% of patients may have adequate dosage due to HA-PCI treatment. But we believe that this is still an acceptable clinical treatment strategy for SCLC. These results provide a theoretical principle in support of investigating the potential benefits of HA-PCI for SCLC.

## Abbreviations

BMs: Brain metastases
GTV: Gross tumor volume
HA: Hippocampal avoidance
HVLT-R: Hopkins verbal learning test-revised
NCF: Neurocognitive function
NSCs: Neural stem cells
PCI: Prophylactic cranial irradiation
PTV: Planning target volume
QOL: Quality of life
SCLC: Small cell lung cancer
SGZ: Subgranular zone
SRS: Stereotactic radiosurgery
SVZ: Subventricular zone
WBRT: Whole brain radiation therapy

## References

[1] Auperin A, Arriagada R, Pignon JP (1999). Prophylactic cranial irradiation for patients with small-cell lung cancer in complete remission. Prophylactic cranial irradiation overview collaborative group. N Engl J Med.

[2] Slotman BJ, Faivre-Finn C, Kramer GW (2007). Prophylactic cranial irradiation in extensive small cell lung cancer. N Engl J Med.

[3] Brown PD, Jaeckle K, Ballman KV (2016). Effect of radiosurgery alone vs radiosurgery with whole brain radiation therapy on cognitive function in patients with 1 to 3 brain metastases: a randomized clinical trail. JAMA.

[4] Gondi V, Paulus R, Bruner DW (2013). Decline in tested and self-reported cognitive functioning after prophylactic cranial irradiation for lung cancer: pooled secondary analysis of radiation therapy oncology group randomized trails 0212 and 0214. Int J Radiat Oncol Biol Phys.

[5] Mizumatsu S, Monje ML, Morhardt DR, Rola R, Palmer TD, Fike JR (2003). Extreme sensitivity of adult neurogenesis to low doses of X-irradiation. Cancer Res.

[6] Suhonen JO, Peterson DA, Ray J, Gage FH (1996). Differentiation of adult hippocampus-derived progenitors into olfactory neurons in vivo. Nature.

[7] Luskin MB (1998). Neuroblasts of the postnatal mammalian forebrain: their phenotype and face. J Neurobiol.

[8] Kundapur V, Ellchuk T, Ahmed S, Gondi V (2015). Risk of hippocampal metastases in small cell lung cancer patients at presentation and after cranial irradiation: a safety profile study for hippocampal sparing during prophylactic or therapeutic cranial irradiation. Int J Radiat Oncol Biol Phys.

[9] Gondi V, Tome W, Marsh J (2010). Estimated risk of perihippocampal disease progression after hippocampal avoidance during whole-brain radiotherapy: safety profile for RTOG0933. Radiother Oncol.

[10] Ghia A, Tome WA, Thomas S (2007). Distribution of brain metastases in relation to the hippocampus: implications for neurocognitive functional preservation. Int J Radiat Oncol Biol Phys.

[11] Marsh JC, Herskovic AM, Gielda BT (2010). Intracranial metastatic disease spares the limbic circuit: a review of 697 metastatic lessions in 107 patients. Int J Radiat Oncol Biol Phy.

[12] Wan JF, Zhang SJ, Wang L, Zhao KL (2013). Implications for preserving neural stem cells in whole brain radiotherapy and prophylactic cranial irradiation: a review of 2270 metastases in 488 patients. J Radiat Res.

[13] Harth S, Abo-Madyan Y, Zhang L (2013). Estimation of intracranial failure risk following hippocampal-sparing whole brain radiotherapy. Radiother Oncol.

[14] Slotman BJ, Mouer ME, Bottomley A (2009). Prophylactic cranial irradiation in extensive disease small-cell lung cancer: short-term health-related quality of life and patient reported symptoms: results of an interrational phase III randomized controlled trail by the EORTC radiation oncology and lung cancer group. J Clin Oncol.

[15] Blomstrand M, Brodin NP, Munck Af Rosenschold P (2012). Estimate clinical benefit of protecting neurogenesis in the developing brain during radiationg therapy for pediatric medulloblastome. Neuro-Oncology.

[16] Marsh JC, Godbole RH, HErskvoic AM, Gielda BT, Turian JV (2010). Sparing of the neural stem compartment during whole-brain radiation therapy:a dosimetric study using helical tomotherapy. Int J Radiat Oncol Biol Phys.

[17] Wang SC, Tang JJ, Sun TT (2017). Survival changes in patients with small cell lung cancer and disparities between different sexes, socioeconomic statuses and ages. Sci Rep.

[18] Schabath MB, Nguyen A, Wlison P, Sommere KR, Thompson ZJ, Chiappori AA (2014). Temporal trends from 1986 to 2008 in overall survival of small cell lung cancer patients. Lung Cancer.

[19] Pesch B, Kendzia B, Gustavsson P (2012). Cigarette smoking and lung cancer—relative risk estimates for the major histological types from a pooled analysis of case-control studies. Int J Cancer.

[20] Varghese AM, Zakowski MF, Yu HA (2014). Small-cell lung cancers in patients who never smoked cigarettes. J Thorac Oncol.

